# LocalView, a database of public meetings for the study of local politics and policy-making in the United States

**DOI:** 10.1038/s41597-023-02044-y

**Published:** 2023-03-15

**Authors:** Soubhik Barari, Tyler Simko

**Affiliations:** grid.38142.3c 000000041936754X Ph.D. Candidate, Department of Government, Harvard University, Cambridge, USA

**Keywords:** Government, Politics

## Abstract

Despite the fundamental importance of American local governments for service provision in areas like education and public health, local policy-making remains difficult and expensive to study at scale due to a lack of centralized data. This article introduces LocalView, the largest existing dataset of real-time local government public meetings–the central policy-making process in local government. In sum, the dataset currently covers 139,616 videos and their corresponding textual and audio transcripts of local government meetings publicly uploaded to YouTube–the world’s largest public video-sharing website–from 1,012 places and 2,861 distinct governments across the United States between 2006–2022. The data are processed, downloaded, cleaned, and publicly disseminated (at localview.net) for analysis across places and over time. We validate this dataset using a variety of methods and demonstrate how it can be used to map local governments’ attention to policy areas of interest. Finally, we discuss how LocalView may be used by journalists, academics, and other users for understanding how local communities deliberate crucial policy questions on topics including climate change, public health, and immigration.

## Background & Summary

Local governments are central to service provision in the United States for policy areas like education, climate change, housing, and public health. Yet, a fundamental barrier stands in the way of a more systematic and empirical understanding of local politics and policy-making: a lack of data. America’s decentralized, federalist system of government makes for both (1) a large number of local governments (nearly 100,000 at last count) and (2) relatively few sources of centralized data on their policies or procedures. Despite constituting the overwhelming majority of elected officials, governing bodies, and political decisions made in the United States, the lack of pre-existing large data sources has limited the scope of the study of local politics based on what data have been feasible for researchers to collect^1–4^. The few large-scale datasets that do exist on local governments, which are generally government releases such as the US Census of Governments for municipalities or the Common Core for school districts from the National Center for Education Statistics, may contain structural and administrative characteristics but are generally insufficient for scholars interested in topics like policy-making and deliberations. A recent explosion of datasets in the social sciences has led to unprecedented, large-scale study of U.S. politics, elections, and policy-making at the national^5–8^ and state levels^9,10^. Meanwhile, most contemporary studies of local policy-making rely primarily on case studies or small sets of individual places^11,12^, lab experiments^13^, or have required extensive (and expensive) manual data collection^14–17^.

Public meetings are the primary policy-making venue for local governments like city councils and school boards. Local officials’ votes on most policies must be taken in public, and open meeting “sunshine laws” in all 50 states generally allow members of the public to pose comments and questions to local officials. However, local government meetings–and local policy-making as a result–are extremely difficult to study at scale. In *City Limits*, a seminal study of local politics, the political scientist Paul Peterson laments that “there is nothing like the Congressional Record”^18^–a complete transcript of proceedings on the House floor–for local politics, making large-scale study of public meetings tedious or impossible for academics, journalists, and members of the public alike. As a result, existing efforts to systematically study local meetings require intense, costly investment in data collection^2,14,16,19^.

Currently, scholars of local policy-making are often tasked with a time-consuming process of manually collecting individual meeting records, often available as meeting “minutes” online. Even once collected, the work is not over, as the lack of any standardized format for published meeting records (e.g. some are direct transcripts, others are summaries; some will include names of public commenters, others will not, etc.) requires difficult decisions about how they should be coded and compared. See Appendix Table A4 for an illustrative example of summarized meeting minutes compared to a transcribed video. For their groundbreaking study of housing and land use politics on planning board meetings^14^, Einstein *et al.* manually collected meeting minutes from 97 cities and towns in Massachusetts between 2015 and 2017, in part due to the uniquely detailed open meeting law in Massachusetts governing written meeting records. Even with funding and time, the lack of standardization in data formats across places creates additional problems, leading others to turn to crowdsourcing collection and cleaning tasks^2^ or lab experiments which simulate local government participation by exposing recruited populations to watch pre-recorded segments^13^.

**Fig. 1:**
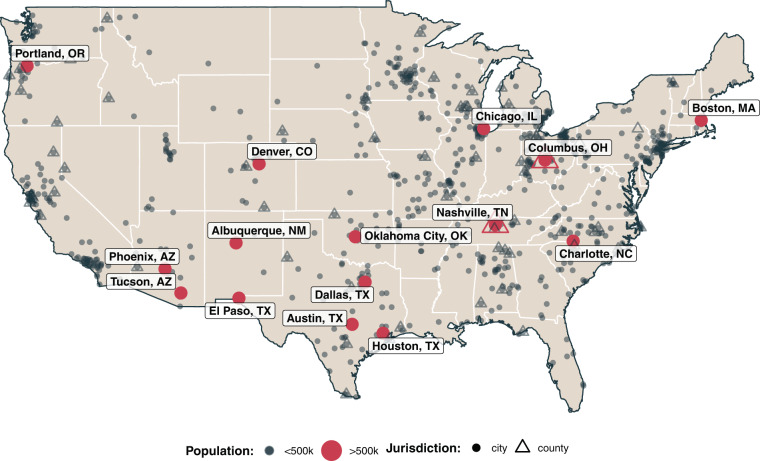
Geographic Distribution of Local Governments in Sample. At present, LocalView covers public meetings held in 2,861 governments across 883 municipal and 129 county jurisdictions in 49 states (all states except Hawaii, which does not have traditional municipal governments and instead leaves the State and four county governments to fill most local government functions). The median population size of a locality in our sample is 13,870 (for reference the median population of a locality in the United States is roughly 1,000). Our sample contains 14 of 33 cities in the United States with populations greater than 500,000. Although the largest cities in the US generally record videos of their public meetings, several (like New York City at the time of writing) rely on private, paid services and not YouTube.

This article introduces LocalView, a dataset of local government public meetings, to aide the study of local policy-making. LocalView is also unique in that it is one of the largest available datasets containing instances of political communication *between* constituents and their government officials, a topic of wide interest in political science^20–23^. Beyond the specifics of US local policy-making, we further believe LocalView can be a useful resource for those studying a number of topics across the social sciences, including the study of deliberative democracy^24–26^, interpersonal communication^27^, and intergroup dynamics along partisan^28,29^, racial^30–32^, geographic^33,34^ or other dimensions. This dataset can aid scholars, journalists, and other observers of local politics and policies in four key ways. First, LocalView allows for the study of local meetings at an unprecedented *scale*. With over 100,000 videos in 49 states, users can explore substantive phenomena of their own interest in a wide range of municipalities and counties (see Fig. 1 for a map of the present coverage). Second, LocalView is unique in its ability to aid analyses over *time*. As described in more detail below, we find that once localities begin posting meeting videos, they largely post all future meeting videos, facilitating analyses that leverage data over many months or years. Third, the *standardization* of meeting transcripts in LocalView can facilitate comparisons across localities. Relying on locally transcribed meeting minutes makes comparison across places difficult due to local transcription idiosyncrasies; LocalView data instead records every word as it was said in the meeting. Finally, our automated data collection and processing pipeline allows LocalView to be a *self-updating* data source. This self-updating feature will expand our coverage both within places over time (as our existing cities post new videos) and across places (as new cities begin posting meeting videos online). Further, these features of LocalView allow it to be of wide interest to scholars using sources of video^13,35,36^, audio^37,38^, and text^39,40^ data in the social sciences.

In the rest of the article, we provide a full description of our data collection, processing, and validation; we demonstrate example analyses and close with discussion of other use cases.

## Methods

**Fig. 2:**
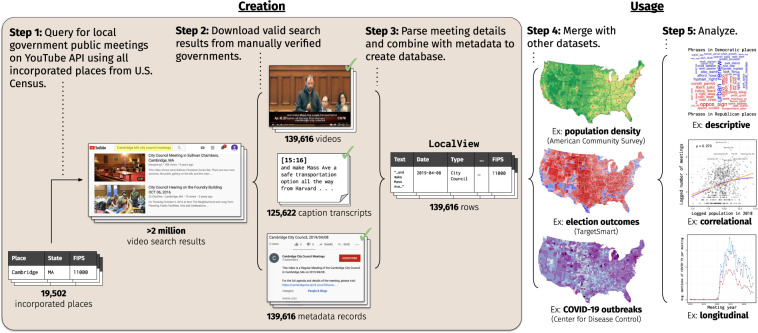
Graphical Overview of Database Creation and Usage. Creation steps are performed on a regular cadence by the maintainers of the database; usage steps demonstrate how users may conduct analyses on the data, once it is downloaded.

Figure 2 summarises how LocalView is created and illustrates some example use cases. This section further details each creation step.

We begin at **Step 1** with a list of all incorporated places from the U.S. Census Bureau. We filter this list to entries with a valid place or county subdivision FIPS code, which are geographic identifiers used widely by the Census Bureau and others to identify geographic places in the United States. We search for each of these entries on YouTube via the YouTube Data API. More specifically, since we do not ex-ante know the type of municipal government in each place, we individually query the place name prepended to each possible municipal government type (e.g., “Jacksonville city council”, “Jacksonville board of selectmen”); later in step 2, we identify the exact government type for each valid meeting video. More than 2 million videos and 2,000 channels are returned by YouTube at this stage.

In **Step 2**, we identify local government channels and public meeting videos from this list of search results. This is done in a sequence of steps at both the channel- and video-levels as follows. First, we manually examine each channel to verify that they post videos about a valid local government (or governments). While we primarily find *municipal* governments–that is, government agencies whose jurisdiction is a locality (not a county) and whose purpose is to serve as the primary legislative body of the locality–we retain channels concerning any local government collected at this stage: county boards, school boards, and other special committees. Since these government bodies’ jurisdictions cover or overlap municipalities in the Step 1 list, they are retained in the final LocalView database.

Next, we manually filter out any invalid channels and videos. Invalid channels are those that post fewer than five meetings, appear to selectively post certain meetings instead of comprehensively posting all meetings, or only post clips of meetings (rather than entire meeting proceedings). Although five is an arbitrary cutoff point, we include a lower threshold for inclusion in the sample as an additional measure of quality control, due in part to the large number of identified instances in which channels upload single videos or clips of videos. We decided to restrict such videos from our sample, as our exploratory research discovered that these individual clips were commonly low-quality and/or edited. Within the set of valid channels in our original search results, we retain only uploads that are public meetings conducted by a local government: that is, they mention both (i) a government name and (ii) a date in either its title or description.

Finally, we identify the exact place, government body, and the type of channel (e.g., officially hosted by the government vs. a broadcasting service or media outlet) associated with each video. This is non-trivial: the search results for each query are not necessarily linked to the original FIPS code searched for. For example, a channel’s uploaded videos may match to multiple municipalities from the Step 1 list or counties and metro areas not in our list (such as a local media outlet that uploads video of city council meetings in multiple towns). To resolve this, we use a combination of string parsing and manual examination to map groups of videos to individual FIPS codes. All videos from a particular channel that mention a particular municipality or county in its title, description, or content are matched to that municipality or county. We then exactly matched each meeting video to the government it represents, supplementing with manual searches on Google and Wikipedia when possible. We similarly use a series of string matches as well as manual audits to categorise (when possible) the channel as an official government channel, a media organization, or a citizen interest group. For more details on sample composition and collection procedures, including a breakdown of government and channel types, see Appendix Section A.

Once the previous verification and identification steps are conducted (see Technical Validation for more details), we download the video files, video metadata (e.g. likes, dislikes, views), and transcribed video caption text (where uploaded by the channel or automatically provided by YouTube) for all valid videos. We rely on YouTube’s transcription algorithm which exhibits a high rate of transcription, roughly 90% of videos. In **Step 3**, this is combined with the parsed information from the previous step (e.g. government type, FIPS code) along with the extracted date for the meeting itself and some commonly available place-level characteristics. We find that meetings are typically uploaded a few days after they are held (roughly 80% of meetings are uploaded less than three days after they were held; 90% within two weeks). See also the Technical Validation section for our manual evaluation of this approach. At this stage, the LocalView database is fully assembled and ready for usage.

## Data Records

The complete LocalView database can be found in the Harvard Dataverse at 10.7910/DVN/NJTBEM^41^. The dataset itself can be accessed in different formats including RDS (to easily interface with the R programming language), Parquet (for efficient data retrieval), .dta (for use with the Stata programming language), .csv, and .json (for easy access with any programming language, such as R, Stata, or Python). Each observation is an individual meeting video.

LocalView comes available as a dataset that can be read in any statistical programming languages that support the formats we provide. No additional software packages or installations are necessary to use LocalView, though researchers may benefit from additional packages that facilitate the kinds of analyses they may perform, such as software for text analysis or mapping. Below, we include a brief example as an illustration for the kinds of analyses a researcher could perform using LocalView.

For features, we store location information including city, state, and FIPS code; the date of the meeting; the date the meeting was posted; the (approximate) date the video was scraped and ingested into the database; YouTube metadata like the URL, channel name; various video-level metadata like the view count, video description, counts of likes, dislikes, favorites, and comments; indicators for whether the video was livestreamed or posted; and the full transcript, where available. Importantly, these are metadata values *at the time of scraping*, which may differ over time (e.g. a video may gain likes over time). As such, future releases of this database will include updated timestamped values for any time-varying fields. When analyzing variables such as likes and dislikes, users should note to incorporate the recording time point of the variable relative to each video’s upload in order to make standardized comparisons. For convenience and as a demonstration of **Step 4** in Fig. 2, we link several political and geographic variables to our dataset (by FIPS code) such as results of several federal and state elections over time and population demographics. As a standardized geographic variable used in datasets like the American Community Survey, we anticipate that FIPS codes will be useful for users to merge LocalView with other datasets of interest. Table 1 displays a relevant subset of these variables along with a description and a real example from the database.

**Table 1:** Selected Columns from Example Entry in LocalView Database.

Variable	Description	Example
st_fips	Concatenated state + FIPS code identifying place of meeting.	4123850
state_name	Name of state.	Oregon
place_name	Name of place as reported in U.S. Census.	Eugene
place_govt	Identified government body.	Municipal Council
place_Pres_dem2pv	2-party Democratic vote-share in last Presidential election.	69.8%
acs_2018_pop	Total population in FIPS area (ACS 2018).	165,997
acs_2018_white	White population in FIPS area (ACS 2018).	138,205
acs_2018_black	Black population in FIPS area (ACS 2018).	2,679
channel_id	URL ID for YouTube channel.	UCW7SKwh_GECGWtH2iPeEaPA
channel_title	Title of YouTube channel.	City of Eugene Public Meetings
channel_type	Who hosts the YouTube channel (when discernible).	Official Government
vid_id	URL ID for YouTube video.	LSN5QBkDtEs
vid_title	Title of YouTube video.	City Council and URA Work Session 06-08-2016
vid_desc	Description text for YouTube video (when scraped).	“Harris Hall, 125 East 8th Avenue”
vid_length_min	Length of YouTube video.	49.98
vid_upload_date	Date of upload to YouTube.	August 12th, 2016
vid_views	Number of views for YouTube video (when scraped).	1
vid_likes	Number of likes for YouTube video (when scraped).	0
caption_text	Caption text for YouTube video, if available, with annotations.	“…we will be holding {00:18:10} {00:18:19} [9 SECOND PAUSE] municipal elections on Tuesday…”
caption_text_clean	Caption text for YouTube video, if available, without annotations.	“…we will be holding municipal elections on Tuesday…”
meeting_date	Date that meeting took place.	June 8th, 2016

Columns omitted from this table for brevity include: further statistics about each video including dislikes and favorites; more population characteristics about each place; results from other federal and state elections in each place; other identifiers about the host of each video’s YouTube channel.

Due to space considerations, we do not include the video and audio recording for each entry. Fortunately, users can easily download the subset of relevant videos or audio recordings for their inquiry via the YouTube API itself using the video or channel identifiers available in LocalView.

## Technical Validation

We discuss the validity of our database along two broad dimensions–*data linkage* (relevant to Steps 1–3 that come from the data creation process Fig. 2 pipeline) and *sample validity* (relevant to Steps 4–5 that are relevant for a particular analysis).

To audit the quality of our data linkage–how accurately individual meeting observations link to other relevant data–we either manually audit a particular variable in its entirety across the dataset or across a reliably large sample when full manual inspection is infeasible. We manually audited all 927 channels to verify (and correct when necessary) the type of host (if it is discernible) and FIPS codes for videos when a channel only maps to a single place. Most of the variables of interest shown in Table 1 and collected in Steps 2–3, however, are video-level so a comprehensive audit was infeasible. Instead, we conduct a randomized audit (*n* = 100 videos) and find the following accuracies: parsed meeting date (93%), government type (91%), municipal or county FIPS code (92%). As an additional sanity check, we examined how keyword counts varied by classified government type. We find keyword counts follow a sensible distribution: compared to a municipal council (the most common government in our sample), the words “zoning” and “planning” are 34% more likely to be mentioned than not in a video explicitly identified as a planning/zoning board, the word “school” 22% more likely to be heard than not in a board of education video, “county” 19% more likely to be heard than not in a county board.

Regarding sample validity, researchers may be concerned with either *internal validity*, or how accurately a channel, place, or government is reflected in our sample or *external validity*, or how accurately the LocalView sample, as a whole, reflects the broader population of interest for a particular analysis. From an internal validity standpoint, the aforementioned audits suggest that the places and governments in our sample are not systematically misidentified; moreover, there do not appear to be any irregularities in counts of channels, videos, metadata records, or transcripts. For example, see the Supplementary Information for various sample size measures over time, which show that LocalView generally grows over time without any abrupt discontinuities due to missingness or other failures on YouTube or the authors’ end. Further, our decision to restrict the sample to channels that upload at least five identified meeting videos means is an additional safeguard against infrequent, low-quality videos uploaded by unofficial actors. Still, there may remain threats to internal validity. For one, the hosts of channels in our sample may still selectively post videos (e.g. a government withholding the posting of a particular video due to a some event at the meeting); though, by removing invalid or likely biased hosts in Step 2 in our pipeline, we believe that this is minimal. Moreover, in supplementary analyses we show that the lack of transcriptions for certain meetings is likely not correlated with regional or populational characteristics (for starters, 90% of our meeting videos have accompanying captions). Recent evaluations of the YouTube transcription algorithm specifically further corroborate that meetings *with* captions are unlikely to have high levels of systemic error^42^.

The external validity of LocalView depends on the analysis a user intends to pursue. As described in the caption of Fig. 1, the size of places we cover, on average, skews larger and many of the largest cities in the U.S. appear to not post their meetings on YouTube nor do many of America’s smallest towns. With our provided identifiers such as the FIPS code, users can weight their analytic sample using common statistical techniques such as raking or post-stratification to more accurately make inferences about the intended population. In the supplementary materials, we demonstrate and discuss how a simple raking algorithm can reduce sample skew on residential population size and key racial demographics (with some caveats). We stress that external validity to the full population of local governments in the United States may not (and should not always) necessarily be the goal of all analyses using LocalView. Instead, LocalView can also be a valuable source of evidence alongside other sources in a mixed-methods analyses (for example, as a database of easily accessible meeting videos for one particular city government of interest). This is to say that the database and approach proposed in this article should supplement, not replace the practices of other, smaller-*n* scholars of local politics^14,18,19^.

## Usage Notes

**Fig. 3:**
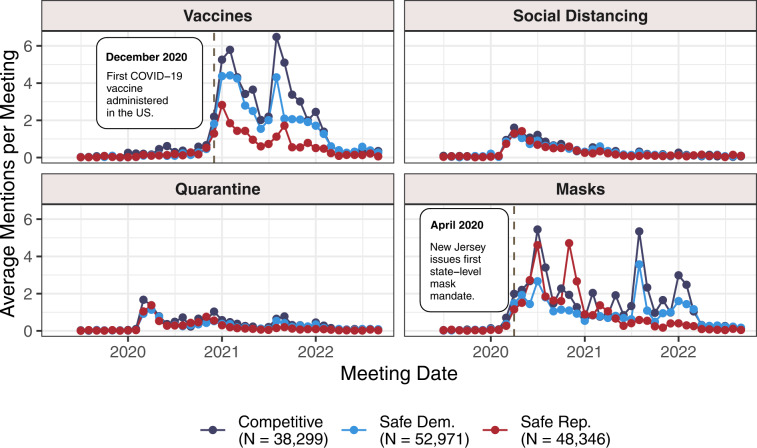
Public Health Policy Deliberation in Different Localities. Classification of municipalities into competitive and safe are done using the average of TargetSmart election results in 2016 & 2018. Safe indicates average party share > = 55%. Meetings are binned to the month, and filtered down to meetings after June 2019. The categories and their respective regular expression strings are Vaccines (vaccin), Social Distancing (social distanc| six feet), Quarantine (lockdown|stay at home|quarantine), and Masking (mask).

As the largest and most diverse dataset of its kind, LocalView is well-suited to aid researchers in describing broad, descriptive patterns concerning different kinds of places across the United States. For example, analysts could explore discussion patterns across particular policy options and places, or how local policy-making discussions change following particular events. In a recent article, Grossman *et al*.^43^ study how political preferences influenced the relationship between governors’ communications about social distancing for COVID-19 and residents’ mobility patterns. The authors use Twitter posts as a proxy for public messages to state government constituents. Conducting a similar analysis on local level policy discussions by governments like city councils or county commissions, where many public health decisions are taken and implemented, would require collecting information from thousands of independent local governments across the United States. Instead, the transcript data in LocalView could be used to measure patterns in local-level deliberations of particular public health policies. For one straightforward measure, users could count the number of times each policy was discussed across places and normalize in some way (e.g. by the number of meetings, words, etc.). As LocalView presents the processed caption data with no additional costly pre-processing necessary, users could count mentions of phrases in any programming language they are familiar with, such as stringr^44^ or quanteda^45^ in R. Figure 3 shows normalized word counts from 2019 through late 2022 for several relevant public health policies: vaccines, social distancing, quarantine/lockdown orders, and masking requirements. Lines are displayed separately by binned categories of average voteshare across two sets of recent elections.

This analysis shows that while social distancing and quarantine discussions appear at similar rates across different partisan contexts, masking and vaccines have much more erratic discussion patterns. Both vaccines and masking follow the general trend of US COVID-19 cases more generally, with peaks in early 2021, late 2021, and early 2022, but general decreased attention throughout the middle of 2021 when cases were lower. LocalView demonstrates that vaccines throughout this time period are consistently discussed less in safely Republican-voting areas than others. This pattern is consistent with Republican hesitancy against vaccines^46^ more generally. Alternatively, attention to masking does not cleanly map onto partisan preferences throughout 2020 and 2021, perhaps illustrating the inconsistencies and confusion around masking requirements that appeared regularly in the US during the onset of the COVID pandemic^47^. However, Republican-leaning areas begin to consistently discuss masking less often starting in mid to late 2021. Attention to masking then shrinks universally across all areas throughout mid and late 2022, coinciding with the return to lower case rates after a spike in early 2022.

Beyond our demonstration here or those illustrated in **Step 5** in Fig. 2, many other analyses may be conducted on salient local issues. Table 2 shows that issues like climate change, affordable housing, crime, gun control, and others are discussed extensively in our meetings (a longer version of this table with more phrases is available in the Supplementary Material). LocalView allows for careful analysis of discussions around topics like gun control, affordable housing and racial discrimination across places and over time **without** extensive and costly manual data collection by researchers. We encourage researchers to fully harness LocalView to both better describe these deliberative trends across time as well as unpack their causes and effects.

**Table 2:** Counts of Salient Phrases in LocalView Database.

Phrase	Total Count	Over Time	Example Quote
**Climate Change**	6,529		**2014-03-25: Issaquah city, WA** Some of these are, as you’ll see very challenging like climate change. I mean that’s a global issue that has some local impacts and we are working. We have a very diligent carbon footprint reduction plan, but are we going to solve climate change? You know, doing our little part probably not, but we can certainly contribute.
**Pandemic**	51,085		**2021-08-18: North Las Vegas, NV**: The good news is we don’t have to create a city of North Las Vegas Health District because we have a very capable health district in our region, but the pandemic has really revealed some deficiencies on our public health infrastructure. Not just for disease surveillance and communicable disease prevention that’s the big ticket item with with the pandemic – but there are other areas in our public health infrastructure that haven’t been strategically invested in over time and they’re deficient and so the health district has been proactive and generated a pretty good strategic plan of their own of how we can regionally enhance public health infrastructure in really meaningful ways throughout our entire community.
**Racism**	11,677		**Salt Lake City, UT, 2018-06-05: I** think we need to take a close look at what has been done and maybe pass some legislation that can make this go away. I feel that the people that have spoke here was about the victimhood of us black males. Not only the black males but the Chicano males that had been affected by this police brutality in this city. I think that you really need to give it a scrutiny so we could curtail it and be a better city because right now Salt Lake City is known as a racist city and you need to understand and each one of you sitting behind there, because they have two killings here and the media has not focused on that. You go to Baltimore, they’re still talking about Freddie Gray. You go to, you go to, you go to St. Louis, they still talking about Mike Brown but we don’t have any echoes.
**Affordable Housing**	41,533		**2016-01-05 Nashville, TN**: We didn’t we didn’t really make a distinction, he never gave us a comparison to other cities about whether our affordable housing issue was exacerbating the amount of rental units as opposed to homeownership, that wasn’t part of the discussion. [What] was part of the discussion was the housing types that are being built across the nation, being very similar to ours, but that being a factor of the recession and millenials living through that and the desirability of homeownership mobility. So that they can move with ease and that the market itself has changed but that’s more universal than it is just to our community.
**Mass Shooting**	177		**2021-04-20 Hutchinson, KS**: You’re invited to pray with me. God of all seasons, as our city is covered with the blanket of snow, we know that you are a blanket of strength covering us through the storms of life. Lord, as a culture we are divided in so many ways: as mass shooting incidences continue to increase, teach us new ways of dealing with frustrations and finding resolutions. Comfort the families of the victims dealing with loss. I lift up our meeting today and ask you God to cover our leaders with wisdom as they work together. Let all voices be heard.

Lines in the “Over Time” column illustrate the relative proportion of meetings that mention topic at least once over the period in our sample (2006–2022).

## Supplementary information

Supplementary Information

## Data Availability

The LocalView dataset is publicly available at 10.7910/DVN/NJTBEM^41^. Code to replicate the main and supplementary analyses in this paper is available at 10.7910/DVN/KHUXIN^48^. More information, including a codebook and related research, is linked on our companion website at https://localview.net.
