# Supplementary material for: LocalView, a database of public meetings for the study of local politics and policy-making in the United States
Source: Sci Data. 2023 Mar 15;10:135. doi: 10.1038/s41597-023-02044-y (PMC10015146; doi:10.1038/s41597-023-02044-y)
Supplement: Supplementary file 1 — Supplementary Information [file 41597_2023_2044_MOESM1_ESM.pdf]

LocalView, a database of public meetings for the study of local politics and policy-making in the United States

Supplementary Information

Contents

A Sample Details **2**
A.1 Meetings By Type . . . 2
A.2 Meetings By Region . . . 3
A.3 Meetings Over Time . . . 5
A.4 Meeting Videos vs. Minutes . . . 6

B Weighting Example **8**

C Phrase Table **10**

A Sample Details

A.1 Meetings By Type

Table A1 provides the break-down of channels and videos in our sample by some commonly occurring, mutually exclusive channel hosts: channels officially hosted by a local government, channels hosted by a media outlet or public broadcasting service, and channels hosted independently (e.g. by a citizen watchdog group or other interest group).

To initially identify official government channels, we search for keywords in the channel title that specify a locality or government (e.g., "city", "county", "municipal", "council") and the keywords "official" or "government" in the channel description. To initially identify media outlets, we similarly string-matched for identifying keywords in the title or description (e.g. "TV", "media", "station", "channel", "reporter") and exclude channels already matched as official. To initially identify independent channels, we use the Named Entity Recognition tool `spaCy` to identify proper nouns in channel names (indicating the possible name of a citizen activist or other individual). From these initial lists, we then manually audited all of the channels' classified types (including those with no classification) and corrected their category. Some channels were easily identifiable due to their extensive descriptions and external URLs to official government websites, online newspapers, blogs, or interest group pages. Other channels' hosts were not discernible or appeared to have been hosted by multiple entities and thus remained unclassified.

We note that these channel types could be further disaggregated: for example, the media channels could be further classified between independent media outlets and public broadcasting services. We leave this to users.

Table A1: **Meeting Channels & Videos by Host of Channel.**

Host Type	Videos	Channels	Videos (%)	Channels (%)
Official Govt	79,078	506	56.64%	54.58%
Media	35,396	199	25.35%	21.47%
Independent	9,685	117	6.94%	12.62%
Unknown	15,457	105	11.07%	11.33%

Table A2 shows the break-down of channels and videos in our sample by government type. The universe of possible names (e.g. "municipal council", "board of trustees") was initially compiled from Wikipedia as well as observing some commonly occurring names in our sample. Some names were aggregated to accommodate idiosyncratic varieties of the same type of body: for example, municipal council includes both city council as well as village council and town council, which are structurally similar but are typically applied to municipalities of different sizes. Others are highly specific (e.g. "Development Corporation") as some channels were entirely or mostly dedicated to very particular local government bodies. As in the classification of channel types, we then string-matched individual videos using their titles and descriptions to these possible names. Additionally, some municipalities' government types are directly available on Wikipedia; for municipal government videos identified from these governments, we directly linked them to their exact government name.

Again, we stress that this is just *one* possible classification scheme for videos in the LocalView dataset. Users may wish to individually categorize governments based on their executive-legislature structure (e.g. mayor-council, city manager) or type of executive (e.g. strong mayor, weak mayor) which may require further hand-coding or merging with an external dataset.

Table A2: **Meeting Channels & Videos by Type of Government**.

Government Type	Videos	Channels	Videos (%)	Channels (%)
Municipal Council	85,646	802	61.34%	29.10%
Municipal Committee	10,626	276	7.61%	10.01%
Planning/Zoning Board/Commission	5,757	264	4.12%	9.58%
School Board	5,320	173	3.81%	6.28%
Other Board	4,640	246	3.32%	8.93%
County Commission	4,564	113	3.27%	4.10%
County Board	4,291	81	3.07%	2.94%
Board Of Selectmen	3,125	35	2.24%	1.27%
Village Board	2,854	40	2.04%	1.45%
Board Of Commissioners	2,229	134	1.60%	4.86%
Board Of Education	2,155	103	1.54%	3.74%
Board Of Aldermen	1,947	24	1.39%	0.87%
County Committee	994	35	0.71%	1.27%
Town Board	910	29	0.65%	1.05%
Board Of Trustees	852	43	0.61%	1.56%
Board Of Health	738	70	0.53%	2.54%
Board Of Supervisors	556	13	0.40%	0.47%
Parks/Rec Board/Commission	408	51	0.29%	1.85%
Special Commission	129	11	0.09%	0.40%
Development Corporation	82	1	0.06%	0.04%
Housing Authority	32	8	0.02%	0.29%
City Commission	4	2	0.00%	0.07%
Unknown	1,757	202	1.26%	7.33%

Notes: Channel %'s do not add up to 100% because channels may have meetings from multiple governments.

A.2 Meetings By Region

Table A3 breaks down the videos in our sample by state. The most represented state in our sample – both by number of videos and channels – is California, while the two least represented are Alaska and North Dakota. At present, our sample does not capture any local governments in Hawaii.

To assess whether there are "accent effects" in the transcription of videos – that is, our dataset capturing fewer captions from localities in certain regions due to accents of speakers – Figure A1 compares different predictors of video transcription per locality across all its YouTube channels

in our sample. Indeed, localities based in the West and the South experience slightly lower rates of captioned videos than those located elsewhere. On the other hand, YouTube's transcription service does not appear to discriminate – at least according to this ecological measure – against less white or more populous localities. Moreover, note that the overall transcription across places in our sample (reflected also in the Intercept term in Figure A1) is 90%. Thus, we find little reason to worry that our sample of meetings is unrepresentative *within place* due to biased transcription errors.

Table A3: **Meeting Channels & Videos by State.**

State:	Videos	Channels	Videos (%)	Channels (%)
Alabama	4,280	32	3.07%	3.43%
Alaska	112	1	0.08%	0.11%
Arizona	2,469	16	1.77%	1.71%
Arkansas	846	12	0.61%	1.29%
California	19,069	78	13.66%	8.36%
Colorado	1,589	18	1.14%	1.93%
Connecticut	512	5	0.37%	0.54%
Florida	904	8	0.65%	0.86%
Georgia	1,021	10	0.73%	1.07%
Idaho	905	4	0.65%	0.43%
Illinois	7,076	49	5.07%	5.25%
Indiana	776	5	0.56%	0.54%
Iowa	1,282	5	0.92%	0.54%
Kansas	330	2	0.24%	0.21%
Kentucky	296	3	0.21%	0.32%
Louisiana	41	2	0.03%	0.21%
Maine	691	5	0.49%	0.54%
Maryland	147	1	0.11%	0.11%
Massachusetts	6,480	16	4.64%	1.71%
Michigan	6,475	47	4.64%	5.04%
Minnesota	5,942	49	4.26%	5.25%
Mississippi	923	7	0.66%	0.75%
Missouri	4,163	40	2.98%	4.29%
Montana	266	6	0.19%	0.64%
Nebraska	1,964	10	1.41%	1.07%
Nevada	340	3	0.24%	0.32%
New Hampshire	1,771	3	1.27%	0.32%
New Jersey	5,333	48	3.82%	5.14%
New Mexico	777	5	0.56%	0.54%
New York	3,678	24	2.63%	2.57%
North Carolina	6,982	51	5.00%	5.47%
North Dakota	5	1	0.00%	0.11%
Ohio	7,574	73	5.43%	7.82%
Oklahoma	3,015	21	2.16%	2.25%

Oregon	4,842	21	3.47%	2.25%
Pennsylvania	3,478	41	2.49%	4.39%
Rhode Island	379	3	0.27%	0.32%
South Carolina	1,657	21	1.19%	2.25%
South Dakota	5,888	12	4.22%	1.29%
Tennessee	4,443	20	3.18%	2.14%
Texas	6,710	63	4.81%	6.75%
Utah	2,531	21	1.81%	2.25%
Vermont	1,264	2	0.91%	0.21%
Virginia	2,816	23	2.02%	2.47%
Washington	3,016	19	2.16%	2.04%
West Virginia	173	5	0.12%	0.54%
Wisconsin	3,770	19	2.70%	2.04%
Wyoming	610	3	0.44%	0.32%

Figure A1: **Predictors of Meeting Video Transcription by Place**

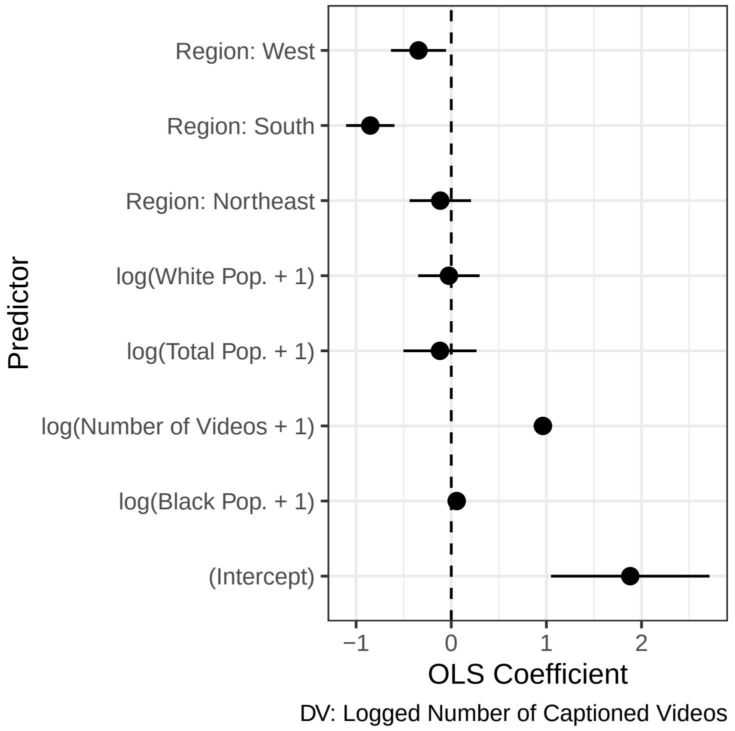

Notes: The baseline region in the model is North Central (i.e. the region more commonly known in the U.S. as the "Midwest").

A.3 Meetings Over Time

Figure A2 visualises how our sample size changes over time across three different units. By all measures, our sample size has grown over time. We hypothesize that the increase in both

videos uploaded by a government and the number of governments itself is due to a confluence of factors: ease of live-streaming videos on YouTube (a feature deployed in 2011 right around the first observed spike in videos and places), decreasing costs of video production, increasing adoption of “digital government” practices by governments, and the widespread transition to entirely online meetings engendered by the COVID-19 pandemic in 2020 (associated with another visible increase in both uploaded videos and governments in the sample).

Figure A2: **Sample Size Over Time**

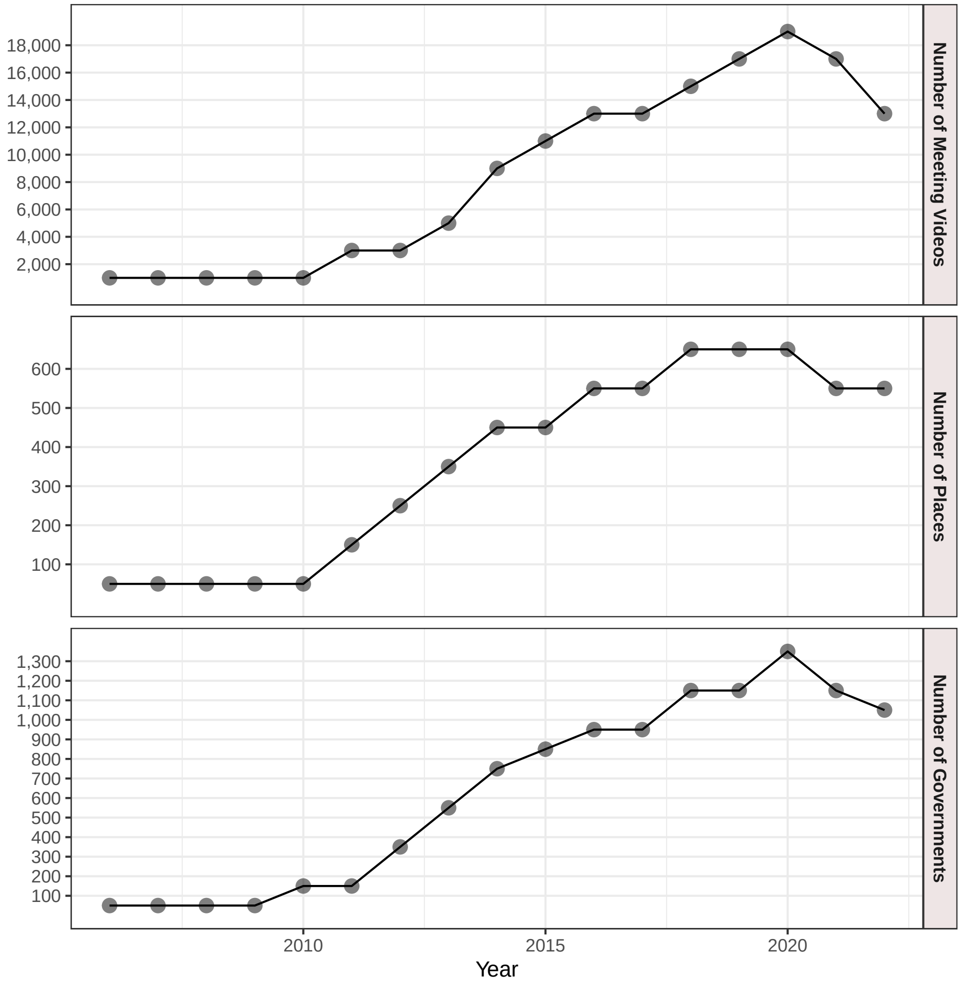

Notes: Meeting videos are counted by the date of *meeting*, not date of *upload*, although a similar trend emerges when using date of upload.

A.4 Meeting Videos vs. Minutes

A commonly available format for local government meeting records is “minutes”, or meeting proceedings either transcribed in real-time or summarised after the fact. As Table A4 shows, minutes often elide important context from the communication and decision-making of constituents or officials. While the minutes simply note that the council member opposed a tax increase, the

video transcript offers insights into the council member's tone, affect, and ideology.

Table A4: **Comparison of Minutes and Transcript of a Public Meeting**.

Minutes	Transcript
Under discussion, Councilmember Kennedy commented that he could not support a **tax increase** which he believes there to be should the bond amendment pass.	Councilmember Kennedy: "Now I know we have some propaganda here that says that there are will be no **tax increase**. Well, this language says the exact opposite. In fact, it mandates the legislature by the state constitution must impose tax rates and therefore I cannot support this ... I don't believe that some people, especially I think the people that designed this, are acting honestly toward the taxpayers. Further, ... if this bond happens and we borrow $1.6 billion and we spend it according to the plan that's been released, ... we're still going to be stuck in the same place where we were before in that we still will not have the money to maintain what we have ... We'll find ourselves in the same hole again but we'll be $1.6 billion further in debt."

Notes: The table compares the minutes and the video transcript for a councilmember's comment in the Clarksburg, WV city council meeting on September 21st, 2017. Clarksburg posts detailed minutes which briefly summarize discussions that happened. Even so, the video transcript reveals important political context for the reasoning behind Kennedy's vote that the minutes lack.

B Weighting Example

One popular method for adjusting sampled data to a population according to observable characteristics so is *raking*.[1] In brief, raking can obtain such weights by iteratively estimating weights that match sample counts of observed variables for which population marginal distributions are available.

Figure B3 (on the next page) demonstrates the results of raking the localities in LocalView along three variables that are skewed – in some cases, considerably – from the census of localities: total residential population, the white residential population, and the black residential population. On the whole, Figure B3 demonstrates that the re-weighted sample is able to accurately resemble the real-world distribution of towns, cities, and counties in the United States along these dimensions.

As in any other case of sample weighting, users should note that subgroups that are exceedingly rare in our sample – for instance, medium sized towns with both relatively large white and relatively large black populations (`Pop (2) x White (2) x Black (2)`) – may be impossible to fully calibrate to the population count.

[1]For a rigorous treatment on the topic, see "Generalized Raking Procedures in Survey Sampling" (Deville, Jean-Claude, Carl-Erik Särndal, and Olivier Sautory).

Figure B3: **LocalView Places vs. Census of Incorporated Places Before and After Raking**

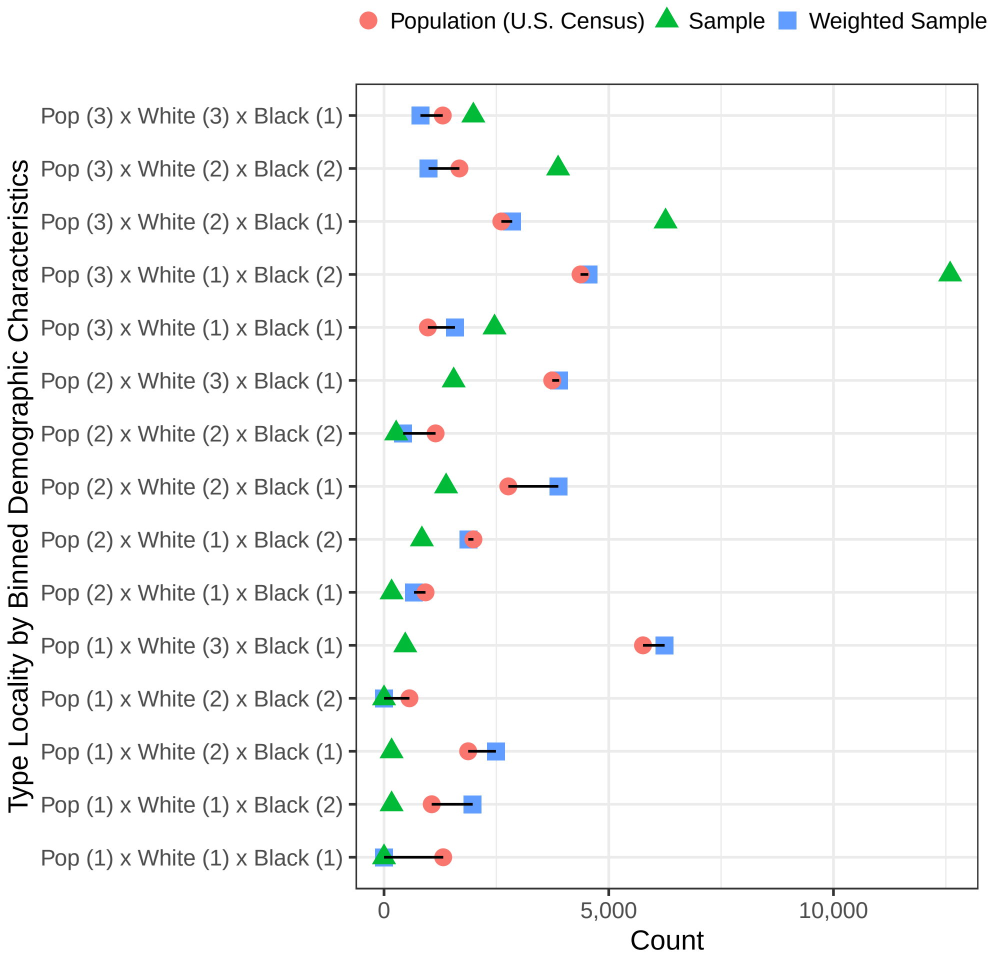

Notes: Horizontal gray lines denote the difference in the population counts (according to the U.S. Census) of each locality group versus the sample count re-weighted by raking. Bins for total population (Pop) are constructed via terciles of the logged population; bins for white and black populations are constructed using percentages relative to the population. On the horizontal axis, the value in pathentheses next to each of the three variables indicates its binned level (e.g. `Pop (1)` is the lowest population size tercile). Subgroups with fewer than 100 localities in the census distribution of localities are not shown in this plot.

C Phrase Table

Table A5: **Counts of Salient Phrases in LocalView Database.**

Phrase	Total Count	Over Time	Example Quote
**Climate Change**	6,529	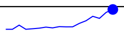	**2014-03-25: Issaquah city, WA** Some of these are, as you'll see very challenging like **climate change**. I mean that's a global issue that has some local impacts and we are working. We have a very diligent carbon footprint reduction plan, but are we going to solve climate change? You know, doing our little part probably not, but we can certainly contribute.
**Pandemic**	51,085	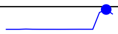	**2021-08-18: North Las Vegas, NV**: The good news is we don't have to create a city of North Las Vegas Health District because we have a very capable health district in our region, but the **pandemic** has really revealed some deficiencies on our public health infrastructure. Not just for disease surveillance and communicable disease prevention that's the big ticket item with with the pandemic — but there are other areas in our public health infrastructure that haven't been strategically invested in over time and they're deficient and so the health district has been proactive and generated a pretty good strategic plan of their own of how we can regionally enhance public health infrastructure in really meaningful ways throughout our entire community.
**Racism**	11,677	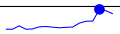	**Salt Lake City, UT, 2018-06-05**: I think we need to take a close look at what has been done and maybe pass some legislation that can make this go away.<br>I feel that the people that have spoke here was about the victimhood of us black males. Not only the black males but the Chicano males that had been affected by this police brutality in this city. I think that you really need to give it a scrutiny so we could curtail it and be a better city because right now Salt Lake City is known as a **racist** city and you need to understand and each one of you sitting behind there, because they have two killings here and the media has not focused on that. You go to Baltimore, they're still talking about Freddie Gray. You go to, you go to, you go to St. Louis, they still talking about Mike Brown but we don't have any echoes.
**Affordable Housing**	41,533	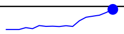	**2016-01-05 Nashville, TN**: We didn't we didn't really make a distinction, he never gave us a comparison to other cities about whether our **affordable housing** issue was exacerbating the amount of rental units as opposed to homeownership, that wasn't part of the discussion. [What] was part of the discussion was the housing types that are being built across the nation, being very similar to ours, but that being a factor of the recession and millenials living through that and the desirability of homeownership mobility. So that they can move with ease and that the market itself has changed but that's more universal than it is just to our community.
**Mass Shooting**	177	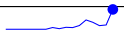	**2021-04-20 Hutchinson, KS**: You're invited to pray with me. God of all seasons, as our city is covered with the blanket of snow, we know that you are a blanket of strength covering us through the storms of life. Lord, as a culture we are divided in so many ways: as **mass shooting** incidences continue to increase, teach us new ways of dealing with frustrations and finding resolutions. Comfort the families of the victims dealing with loss. I lift up our meeting today and ask you God to cover our leaders with wisdom as they work together. Let all voices be heard.
**Vote By Mail**	736	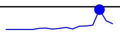	**2021-10-12 Howell Township, NJ**: Today is the deadline to register to vote, so if you're not registered please go on monmouthcountyvotes.com.<br>There's three ways to cast your ballots. Right now it's **vote by mail**, early in-person, or at your polling location so today is the last day.<br>So if you could, if you're not registered to vote please do so.

**Minimum Wage**	3,514	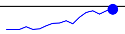	**2016-08-23 Mobile, AL** I'm with a group called the Alabama Coalition for Economic Justice. We were active in Birmingham and passing a **minimum wage** of ten dollars and ten cents that was subsequently rescinded by the state, but it granted a raise to 42,000 people before they did that.
**Sanctuary City**	326	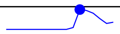	**2020-06-17 Boston, MA** As a country, we have seen the many changing attitudes towards refugees in our domestic and international policies throughout our history. This day, particularly during this pandemic gives us an opportunity to seriously consider the hardships and danger that would lead to people becoming refugees. It gives us an opportunity to remember why we are a **sanctuary city** and why Boston is strengthened by its immigrant communities, and why we must continue to remove every barrier that makes living here harder than it should be, particularly for our refugees.
**Gun Control**	3,380	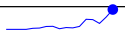	**2018-10-15 Stanly County, NC**: The timing is bad in my opinion. We are 13 days before gun season, 11 days before early voting and 22 days before the November elections. To be putting up a **gun control** ordinance 22 days before the fall elections, I think puts a lot of our candidates in a very unsavory spot. I wouldn't want to be them. Luckily I'm not I won my seat, I'm ok, but this is not a question that I want to be answering to snap tomorrow. Yet, this goes through there's been to my knowledge no input from the Stanly County Sheriff's Office. I think law enforcement needs to be consulted on this. Law enforcement is going to be the one that has to go out and be the boots on the ground.
**Crime**	37,423	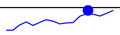	**2016-02-08 Bellbrook, OH**: The **crime stats**, the breakdown as you can see — the biggest several that we have, number one is ID theft and I'll get to that here in just a little bit. Domestics and drug-related crimes, not that shouldn't alarm everybody that because we only had 25 drug-related offenses. I don't want to be an alarmist that we have a drug problem. Sure, we, it's relative to other cities and what they're facing. You may have seen things from the news recently with the heroin epidemic. Do we have heroin in the city? Yes. Is it an epidemic in the city? No. Is the problem with certain people? Yes. Do we know about it and are addressing it? Yes.
**Inflation**	11,316	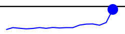	**2016-09-19 Joliet, IL**: Water and sewer rates have been dramatically outpacing **inflation** when it comes to cost escalation, really since the mid-80s. I stopped this chart at 2000. The last item to note here is the change in Joliet rates. So the green line represents the changes that have been made to Joliet residential rates since 2000, and you'll see some activity, pretty substantial increases in 2009 and 2011, but rates have been flat, unchanged for your community since then. Let's talk just a moment about some of the factors that contribute to those rising rates.

Notes: Lines in the "Over Time" column illustrate the relative proportion of meetings that mention topic at least once over the period in our sample.
